# “Follow the Musical Road”: Selecting Appropriate Music Experiences for People with Dementia Living in the Community

**DOI:** 10.3390/ijerph20105818

**Published:** 2023-05-13

**Authors:** Lisa Kelly, Amy Clements-Cortés, Bill Ahessy, Ita Richardson, Hilary Moss

**Affiliations:** 1 Health Research Institute, University of Limerick, V94 T9PX Limerick, Ireland; 2 Lero—The Science Foundation Ireland Research Centre for Software, University of Limerick, V94 T9PX Limerick, Ireland; 3 Faculty of Music, University of Toronto, Toronto, ON M5S 1A1, Canada; 4 Health Service Executive, D08 K3V9 Dublin, Ireland

**Keywords:** music experiences, music-based interventions, music therapy, dementia, community

## Abstract

There are many music experiences for people with dementia and their caregivers including but not limited to individualized playlists, music and singing groups, dementia-inclusive choirs and concerts, and music therapy. While the benefits of these music experiences have been well documented, an understanding of the differences between them is often absent. However, knowledge of and distinction between these experiences are crucial to people with dementia and their family members, caregivers, and health practitioners to ensure a comprehensive music approach to dementia care is provided. Considering the array of music experiences available, choosing the most appropriate music experience can be challenging. This is an exploratory phenomenological study with significant Public and Patient Involvement (PPI). Through consultation with PPI contributors with dementia via an online focus group and senior music therapists working in dementia care via online semi-structured interviews, this paper aims to identify these distinctions and to address this challenge by providing a visual step-by-step guide. This guide can be consulted when choosing an appropriate music experience for a person with dementia living in the community.

## 1. Introduction

Dementia is a syndrome, or set of cognitive and behavioral symptoms, in which multiple-domain cognitive impairment is sufficiently severe to affect everyday function [1]. This may include the impairment of memory, thinking, orientation, comprehension, calculation, capacity for learning, language skills, and judgment [2]. There are over 100 diagnostic categories of dementia [1]; however, four main types account for 90–95% of cases, the most common being Alzheimer’s disease, which accounts for 60–70% of cases globally [3]. Other major forms of dementia include vascular dementia as a result of stroke [4], dementia with Lewy bodies [5], and frontotemporal dementia [6]. Depending on the underlying causes of dementia and the impact of other health conditions, the signs and symptoms of dementia are unique to each individual and often do not occur in a linear fashion.

It is estimated that there are 55 million people with dementia globally [3]. This figure is expected to significantly rise to 78 million in 2030 and nearly double to 139 million by 2050. The World Health Organization recognized dementia as a public health priority in 2017, endorsing the *Global action plan on the public health response to dementia 2017–2025* [7]. The majority of people with dementia live at home with support from family and/or informal caregivers. With the right supports and social connections, people with dementia can live well in the community for a long time. As a result, enhanced community supports and understanding about how best to ensure people with dementia are socially connected is a priority for policy and planning in this area [8].

There has been an increased interest in non-pharmacological approaches, including the arts, to increase health and well-being for people with dementia [9,10]. The use of music in this regard has been associated with several health benefits, including improved communication, mood, and memory; reduced behavioral and psychological symptoms; and an overall enhanced quality of life [11]. Music in dementia care is delivered through a “continuum” [12] of experiences, including music therapy [13,14] and a variety of music experiences [15], which will be the focus of this paper.

This study is situated in the context of a larger “parent-study”, which explored how telehealth music therapy can support people with dementia and their family caregivers in Ireland. Telehealth music therapy refers to the synchronous delivery of music therapy using pre-existing audio–visual telecommunications technology. Guided by people with dementia through Public and Patient Involvement (PPI), the interdisciplinary research project has multiple research objectives, which aim to explore how person-centered dementia care can be provided in music therapy [16], the music and technological considerations when designing a telehealth music therapy program for people with dementia [17], music therapists’ experiences of providing telehealth music therapy for people with dementia [18], and the experiences of persons with dementia and their spouses who attended a telehealth music therapy program [19]. It became apparent that, before we could consider how telehealth music therapy could support this population, there was a need to (a) identify what music experiences were available for people with dementia and (b) develop an understanding of how to determine which music experience may be most appropriate to meet the psychosocial needs of people with dementia and their family caregivers in the form of a practical guide. Consequently, this is the research focus of this current paper.

### 1.1. Music in Dementia Care—A Continuum of Experiences

A wide array of music experiences exist for people with dementia, their families and caregivers in the community, and in healthcare and residential settings. The distinction between these music experiences, whether therapeutic or recreational, is often blurred. The recent expansion of music-based interventions and music therapy practice for people with dementia raises numerous questions. Most importantly, how do we differentiate between music therapy, which has a clinical focus, and other music-based interventions or experiences, which have a recreational or well-being focus and are delivered by musicians, music and health workers, caregivers, or health professionals? [20].

Music experiences exist on a continuum of recreational to therapeutic opportunities [12]. Some experiences afford the capacity to meet both therapeutic and recreational aims, which are targeted simultaneously. Music experiences can also be active or receptive in nature. Active music making refers to playing instruments, songwriting, singing, and movement to music, while receptive music making refers to music listening and music for relaxation. Confusion regarding the identification and terminology of music experiences in healthcare settings is commonplace and has been addressed by researchers [12,21,22]. In fact, many music experiences in medical settings are often referred to as “music therapy” when this is not the case [23]. In the development of a new arts and health paradigm, Moss [22] described the various applications of music in healthcare, including music therapy, music educators, music listening, musicians in residence, music and health practitioners, community musicians, live performances, and music as part of a building design (aesthetics). The author provides a comprehensive model to understand how the arts (in this case, music) can be used within healthcare settings. It acts as a useful model to consider the music experiences available to people with dementia. Music medicine is another specialized area of work usually administered by healthcare professionals other than music therapists and often focusing on the clinical effects of music through passive music listening programs [24,25].

Music activities delivered by people who do not have a formal music therapy qualification generally tend to be focused upon the following: public performances curated for people with dementia; individually compiled playlists; music appreciation; entertainment with a focus on interactive engagement (joining in singing, playing percussion instruments); karaoke; songwriting workshops; and dementia-inclusive choirs, which can be led by music therapists or non-music therapists [26,27,28]. There is also a significant benefit in allied healthcare professionals and caregivers using music at other times to facilitate music experiences for a person with dementia.

Accessible music experiences are often not resources that are available to many people with dementia living in the community or those caring for them [29]. The online delivery of these experiences offers increased access to those who cannot access them in person [30] and has the potential to diminish the disparity of care for people with dementia living in rural and remote areas [31]. Several studies report on music experiences being delivered online for people with dementia, including dementia-inclusive group singing [30] and music therapy [32,33]. Each of the aforementioned music experiences provides unique benefits at various stages of the disease trajectory.

### 1.2. The Musical Care Pathway—A Useful Model

An understanding of the various music opportunities is crucial to family members of people with dementia, caregivers, and health practitioners to ensure a comprehensive and holistic approach to care is provided. The Musical Care Pathway was published in 2021 as part of the Music for Dementia Campaign (https://musicfordementia.org.uk/wp-content/uploads/2021/03/Musical-Dementia-Care-Pathway.pdf (accessed on 3 May 2023)), an initiative of The Utley Foundation, a grant-making charitable foundation in the UK [34]. The creative representation of the graphic entitled “Our Journey With Music Through Dementia” is a useful resource, which highlights how music can be woven into care across the trajectory of the disease. It illustrates the diverse settings where music can be used, including at home, community settings, places of worship, arts venues, hospitals and clinics, and various care settings, including nursing homes, retirement residences, and assisted living. The ways music can be utilized are also presented and include recreational experiences, music therapy, and music experiences, which might happen in more than one category. These include listening to music, learning an instrument, singing in a group or choir, participating in music groups, and music therapy.

However, difficulty in choosing the most appropriate music experience can be a challenge, especially considering the array of music experiences available. Subsequently, an increased cognitive decline and reduced mobility are inherently associated with the progressive nature of dementia, which creates potential accessibility issues for some people with dementia living in the community. This paper aims to address these challenges by providing a guide that can be consulted when choosing an appropriate music experience for a person with dementia. “Follow the Musical Road: A guide to choosing a music experience for people with dementia living in the community” offers a step-by-step visual map to aid people with dementia living in the community, along with their caregivers, supporters, and health practitioners, to decide which music experience may be of most benefit to them. The pathway presented below was designed based on the findings of an extensive literature search, in consultation with PPI contributors and music therapy experts. It considers both those who have access to in-person community support services and those who do not. This paper begins with an overview of the various music experiences for people with dementia, before describing the research and development of the new guide.

## 2. Literature Review

The focus of this study is on music experiences in the community or home setting for people with dementia. Therefore, music therapy and music-based intervention studies in this context were the focus of this review. The search was limited to English language studies from 2000 onward. Electronic databases searched included PubMed, Sage, ScienceDirect, and Web of Science. The combinations of search terms used are presented in Table 1. A hand search of non-ISI journals relating to music therapy and/or music and health for this population including *Music Therapy Perspectives*, *Music and Medicine*, and *Voices: A World Forum for Music Therapy* was also completed. Following a comprehensive literature search, each of the music experiences and their associated benefits will be described in turn.

### 2.1. Music Listening

Music listening is an everyday leisure activity for older adults, linked to improved psychological well-being and positive emotional responses [35]. Evidence suggests that music engagement leads to an improved quality of life for many older adults [36]. The effects of individually tailored and personalized recorded music or playlists for people with dementia have been widely explored in the literature [23,37,38,39,40]. The use of personalized playlists holds some promise as a useful alternative to music therapy or live music performances as it is relatively inexpensive and increases the accessibility of pre-recorded music [23]. However, evidence regarding the efficacy of music listening interventions have produced mixed results, warranting the need for more methodologically rigorous studies to determine their effectiveness [41]. The use of personalized playlists rather than listening to music collectively in a group generally appears to be the optimum method of using recorded music with older adults in residential care, with reducing agitation being the most prominently observed benefit [23]. This may be because group listening experiences do not cater for the individual music tastes of those listening [42]. As well as catering for individual music preferences, the mental health history and symptoms of the person with dementia must be taken into consideration as individual differences amongst participants have been observed [38]. It is imperative to have appropriate support systems in place and choose music with care when using music with vulnerable people with a history of or current symptoms of depression [37]. For some people with dementia, music listening has demonstrated a positive effect and can improve symptoms of sundowning, reduce neuropsychiatric symptoms, and lower levels of agitation [40]. Preferred music can also be a valuable tool in assisting people living with dementia to connect with their identity and life history, acting as a springboard for reminiscence [37,43]. Personalized playlists can also be used as part of music therapy programs to support the client between sessions.

### 2.2. Dementia-Friendly Concerts

Shibazaki and Marshall [44] explore the various ways live music concerts can influence people with dementia, their caregivers, and their family members. The study, which took place in both the UK and Japan, explored the impact that a series of music concerts held in care facilities had on the people living with dementia, their nursing staff, and their families. A total of 22 concerts were held: 11 in the UK and 11 in Japan. Each concert was for the duration of one hour and contained a similar style of repertoire, including instrumental music for listening as well as familiar songs/pieces, which residents could sing along to if they wished. Interviews were carried out with participants immediately after the concert ended, with questions covering feelings relating to the concert, overall musical preferences, the impact of the music, and reasons for attending the concert. Congruent with findings from previous studies, participants demonstrated the ability to remember musical content and words of songs from a wide array of genres that spanned multiple decades [45,46,47]. As well as this, increased levels of communication between participants were observed following the performances [44]. It was also noted that a small number of participants demonstrated increased agitation or a loss of interest.

### 2.3. Continuing to Play/Learning to Play an Instrument

Playing a musical instrument is a common leisure activity that can promote positive cognitive effects and has been demonstrated to have a positive impact on mental health [48,49]. Furthermore, Baird and Thompson [50] propose that playing a musical instrument allows a unique form of access to engaging memory and promoting a strong sense of self. It is a highly complex skill, which activates numerous neural networks, including primary sensory and motor regions, association areas, frontal brain regions, and emotional and memory networks. Preliminary evidence suggests that playing a musical instrument is associated with a decreased risk of dementia [51,52] and slower cognitive decline [53]. Music lessons are more commonly offered to those who have previously played a musical instrument with the aim of reactivating a learned skill or encouraging a person with dementia to continue to play an instrument despite a cognitive decline [54]. For those who do not play a musical instrument, a singing group may be an alternative option.

### 2.4. Group Singing and Dementia-Inclusive Choirs for People with Dementia and Their Caregivers

Music activities, such as group singing, can improve the well-being and quality of life of people with dementia, modulating mood, reducing symptoms of anxiety and depression, and enhancing awareness and cognitive function [55,56]. In addition, group singing may be beneficial to healthy ageing and may promote respiratory muscle strength in older adults [57]. Lee et al. [29] investigated community group singing to promote well-being for people with early-stage dementia and their family caregivers. Participant feedback indicated that the roles of caregiver and care-receiver became indistinguishable and that the sessions enabled them to spend meaningful time with their family member. The authors suggest that community group singing for people at the same stage of the disease trajectory and their family caregivers can promote feelings of solidarity, support, and belonging. It appeared that the sessions also contributed towards a sense of purpose for the participants at the early stage of their dementia diagnosis, providing them with something to look forward to and prepare for. Similarly, other studies have reported that community therapeutic group singing has been used to support people with dementia and their caregivers, fostering acceptance, improved social confidence, a positive mood, reduced anxiety, reduced pain, and purpose [58,59,60].

Choir singing is an accessible and enjoyable music activity that can support the health and well-being of people with dementia and their family caregivers [61]. Dementia-inclusive choirs promote social inclusion and aim to reduce the stigma associated with a diagnosis of dementia. Mittelman and Papayannopoulou [62] reported that people with dementia who participated in a dementia-inclusive choir alongside their caregiver experienced significant improvement in quality-of-life measures and a positive impact on communication with their caregivers. At the later stages of dementia, participation in a music or a singing group may not be possible due to an increased cognitive decline or reduced mobility. Additionally, communication can become impaired, making meaningful interaction with others difficult. At this point, music therapy may be beneficial.

### 2.5. Music Therapy

Music therapists use the unique qualities of music and the development of a therapeutic relationship to access emotions and associated memories, structure behavior, and to provide social experiences to promote communication and social interaction in order to address clinical non-musical goals [63]. Music therapy interventions for people with dementia typically include community music therapy groups with caregivers and their families; group music therapy in residential care and hospitals for people at different stages of dementia; and individual music therapy [20]. Additionally, music therapy can also be delivered via telehealth for people with dementia [32,33] from the comfort and security of their home.

There is an increasing body of evidence supporting the use of music therapy with older adults as it provides creative, non-pharmacological, and cost-effective programming, in both residential care and in the community [64]. There are times when music therapy may provide optimum benefits for a person with dementia and is indicated above the inclusion of all other music experiences [23]. The unique potential of music therapy for people with dementia includes many features, such as offering non-verbal possibilities, the engagement of all the senses, and offering opportunities for artistic spontaneity and musical narrative, as well as the physical, intellectual, and emotional capacity of music [20]. Methods used in music therapy with this population may include song singing [65], improvisation [66,67], songwriting [68], reminiscence-focused music therapy [69], and music listening or receptive techniques [70].

“Person-centered music therapy,” synonymous with best practice in dementia care, focuses on meeting Kitwood’s [71] core psychological needs of people with dementia (attachment, comfort, inclusion, identity, and occupation) through the therapeutic relationship that develops [68,72,73,74]. The music therapist plays a unique role in dementia care settings, providing interventions where other professionals or caregivers might not easily meet specific needs for people with dementia and their caregivers [20], for example, when a person experiences behavioral and psychological symptoms in the later stages of dementia or when cognitive function deteriorates and confusion is prevalent. Music therapy is also used in dementia and palliative care and can offer opportunities to reduce isolation, lift mood, and express spirituality [46,75].

## 3. Methods

### 3.1. Research Approach

This is an exploratory phenomenological study with significant PPI. As we were interested in understanding the lived experience of the PPI contributors’, phenomenology was deemed the most suitable qualitative model as it takes an idiographic perspective, is concerned with representing the essence of the lived experience of the participants [76], and can also expand professional knowledge [77]. Contrary to objectivist research that seeks to test hypotheses or specific theories, phenomenological research operates from a constructionist epistemology, which aims to promote discovery and description [78].

PPI refers to “doing research with or by the public, rather than to, about, or for them” [79], pg. 6. In health research, PPI is becoming increasingly popular due to the growing recognition that it has the capacity to increase the relevance and utility of research outputs to the population in question and the public while ensuring research deliverables that can be recognized as relevant to and connected with people’s lives and well-being [80,81,82]. The involvement of people with a diagnosis of dementia in PPI and engagement in research design and delivery is an emerging field [83] and has been recommended by Alzheimer Europe [84].

### 3.2. Participants

Seven participants were involved in this study. Three were PPI contributors and four were key informants who are music therapists. The PPI contributors in this project are three people (one female/two male) with varying diagnoses of dementia (early onset/Alzheimer’s disease/Lewy Body Dementia) who are living at home. Each of the PPI contributors are members of the Dementia Research Advisory Team at The Alzheimer Society of Ireland and were recruited via this organization.

The key informants were music therapists (three female/one male) with over twenty years’ experience working as a music therapist who have worked extensively in dementia care settings. They were recruited via email between June and August 2022. Purposive sampling was used when recruiting the key informants to ensure the selection of information-rich cases [85]. Each of the participants were professional contacts of the first author. The inclusion criteria were as follows: (a) fully qualified music therapist or equivalent in the area where they resided with a minimum of five years’ experience working with people with dementia and (b) the participant must read, speak, and understand English.

### 3.3. Research Method

Data were collected in two phases: (1) an online focus group with PPI contributors with dementia and (2) online semi-structured interviews with key informants who are music therapists. The collection of qualitative data in an online format is advantageous due to the reduced travel cost, time efficiency, and the fact that it provides additional time and flexibility for participants to reflect on and respond to the discussed topics [86]. Furthermore, Guest et al. [87] found that online modalities for conducting qualitative research did not result in substantial or significantly different thematic findings than in-person data collection. They also enable the recruitment of demographically and geographically diverse participants [88].

Phenomenological researchers mainly collect data via individual in-depth, semi-structured interviews to obtain a description of the lived experience of a particular phenomenon. Open-ended questions are used to encourage participants to reflect on their experiences from multiple perspectives [78]. Traditionally, focus groups are not associated with phenomenological research and have been highlighted as “incompatible” by some [89]. Others have argued for the place of focus groups in phenomenological research and have suggested that the individual voice of participants can be preserved and used as a springboard for the interaction afforded by the focus group interview [90]. We recognize that individual interviews with the PPI contributors could have made it possible to explore their experiences in more detail. However, the broad and exploratory nature of questions posed in phenomenological interviews are not always accessible for people living with dementia [91]. The focus group setting provided a supportive framework for the PPI contributors to mutually reflect upon the questions asked and hear the ideas of others, which in turn helped them to formulate their own opinions [92]. This method has also been successfully used by other music therapy researchers following a phenomenological approach [93].

### 3.4. Ethics

Ethical approval was sought and obtained through the Research Ethics Committee at The University of Limerick. When recruiting the PPI contributors, a research officer was assigned through the Alzheimer Society of Ireland to ensure the safety of the contributors. An information letter and consent form were distributed to all participants pre-interview. Informed consent was obtained in both written and verbal format prior to the research.

### 3.5. Research Design

#### 3.5.1. Phase One: Focus Group with PPI Participants

An online focus group meeting was held to establish the focus of the research and key questions to be answered. Due to COVID-19 restrictions, the online focus group, which included members of the research team and the PPI contributors, was held on Zoom for 60 min. The aim of the focus group was to collect data using guided group discussion to gather a rich understanding of the participants’ lived experiences and beliefs. The discussions were moderated by the first author, who did not directly participate in the discussion itself but raised questions and encouraged the PPI contributors to reflect on topics openly.

Subsequently, the first author researched and developed Draft 1 of “Follow the Musical Road”, which was informed by an extensive literature search and the research priorities determined in Phase One by the PPI contributors. Following its development, the first author returned to members of the research team (Authors 3–5) with the preliminary design for review and suggested edits.

#### 3.5.2. Phase Two: Semi-Structured Interviews with Music Therapists

The revised document was then circulated via email to four key informants (a non-random selection of international experts (senior music therapists) who have worked extensively in dementia care). Semi-structured interviews were conducted with the four key informants. Due to the widespread geographical dispersion of participants, interviews were facilitated on Zoom and audio-recorded using the record function on the application. The music therapists were asked four open-ended questions:

- How do you see this document being used?
- Can you see this being utilized more by music therapists, other professionals or people living with dementia and their family caregivers?
- Do you think that there is anything that should be changed/added/removed?
- Is there anything that you think doesn’t make sense?

Post-interview, transcriptions of the recordings were produced for analysis and, subsequently, the audio files were permanently deleted. The data from Phase Two were analyzed by Author 1 and suggested edits were made, resulting in the final draft of “Follow the Musical Road” being produced.

### 3.6. Data Analysis

Responses from the individual online interviews with the key informants were transcribed verbatim into Microsoft Word. Data were analyzed using thematic analysis based on a descriptive phenomenology [94], a process that is guided by the methodological principles of “emphasizing openness, questioning pre-understanding and adopting a reflective attitude” (p. 735). This process followed three main steps: (1) achieve familiarity with the data through open-minded reading, (2) search for meanings of lived experiences and emerging themes, and (3) organize the meanings into patterns and write the results of the themes related to the study aim and the context of the research.

## 4. Results

### 4.1. Phase 1

Four research priorities were determined with the PPI contributors and underpinned the development of the guide presented in this paper:

1. The identification of the various music experiences and supports for people with dementia.
2. How does a person with dementia living at home and/or their family caregiver determine what music experience may suit them best?
3. The need for a person-centered approach to be adopted when designing services for people with dementia.
4. To provide psychosocial support services to people with dementia who are unable to access community support services in person via telehealth.

The first draft of “Follow the Musical Road” can be viewed in Figure S1.

### 4.2. Phase Two

Suggestions from the key informants included the use of graphics as well as text to make the document more accessible for those who might have difficulty reading due to cognitive decline: *I think particularly when we’re working with people with dementia, anything that can engage the senses and communicate in multimodal ways that doesn’t always rely on language and text could be helpful* (MT1).

The issue was raised of whether the music experiences outlined in the document would be available to the person with dementia, family member, or support worker using it and whether this would be “misleading” (MT4). It was suggested that perhaps the document could be customizable depending on the location it is being used in and the support services available in the area.

One of the key informants suggested that the document could be useful for music therapists in supporting people with dementia and their families in a person-centered way:

*So, it is almost like a road map that can help the music therapist guide the conversation to help the caregiver and the person with dementia determine what suits them, so I think in a lot of ways it’s very person centered, and it gives agency. You are not just throwing out lots of ideas which could be overwhelming, but rather taking it step by step. “You have these resources. Do you think you might benefit from more resources?”.*

(MT3)

Person-centered language was also highlighted as important. MT4 suggested that person-first language should be used if the document will be used by a person with dementia rather than third-person language:

*If it was going to a person living with dementia you would not want to be asking ‘do they need’. There would be a ‘you’, ‘do you’ or ‘are you’ in there [the document]. So, there would just be that kind of change in language.*

### 4.3. The Final Draft

**Figure 1:**
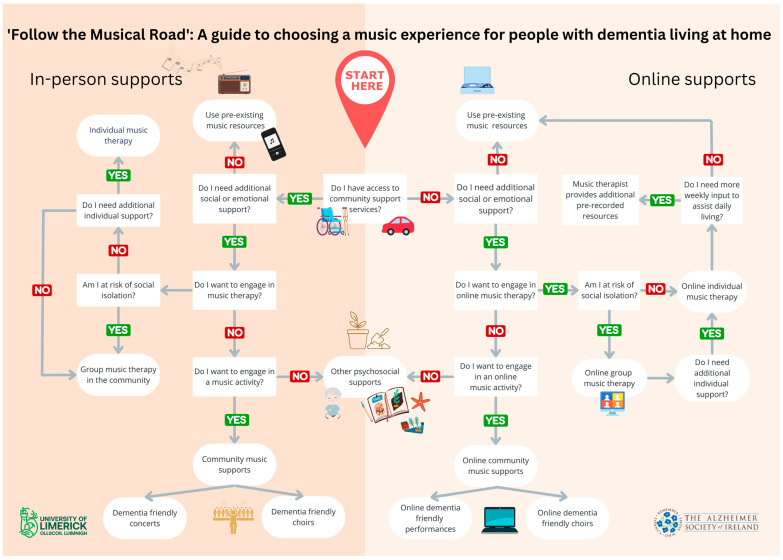
Final Draft of “Follow the Musical Road”: A guide to choosing a music experience for people with dementia living at home.

The final guide (Figure 1) takes the form of a decision tree, guiding the reader through a series of “yes” or “no” questions to determine what music experience might suit them given their circumstances. First-person language was used to ensure the document was person-centered and to promote a sense of agency. The first question discerns whether the reader has access to community support services. A person with dementia may not have access to community supports for several reasons, including reduced mobility, a lack of access to transport, an increased cognitive decline, or because they live in a rural area. From here, the decision tree breaks into two main sections: in-person supports and online supports.

The second question was to determine whether psychosocial support is needed. This may include supporting the person with dementia to ensure their mental, emotional, social, and spiritual needs are met. The subsequent questions focus on determining what the person with dementia themselves would like to engage in. Autonomy and choice are essential in choosing which support might be best suited and individual preference should be respected and encouraged.

## 5. Discussion

The presented guide is not an attempt to rigidly decide which music experience a person with dementia must avail of but rather acts as what Bonde [95] describes as a “descriptive and metaphorical endeavor” to create an “orientation tool”, which aims to map the terrain of music experiences for people with dementia (p. 133). We view the guide presented in this paper as a discussion tool to open up the conversation regarding a person with dementia’s needs and what supports might be most suitable while limiting confusion of different interventions or experiences in a rapidly expanding music and health era.

The guide may be useful in three contexts: It may enable a conversation between the person with dementia and their family member or supporter about what music experience may be preferable or suitable. It may act as a useful tool for music therapists to use in the referral and assessment stages to determine the needs of the client and availability of services. Lastly, it may provide a comprehensive overview for GPs, dementia case managers, dementia advisors, and other healthcare professionals of the various ways music can be utilized over the course of the dementia trajectory. The inclusion of “other psychosocial supports” was also deemed important as we acknowledge not all people may have an interest in music.

One may argue that the inclusion of both the in-person and online branches of the diagram is unnecessary given that they more or less follow the same structure. The inclusion of the dual modes of delivery aims to highlight that by harnessing the functions of pre-existing audio-visual technologies and delivering synchronous music experiences to this population online we can provide an all-inclusive model of music experiences for people with dementia living in the community. This has the potential to increase access to those who may no longer be able to attend community support services due to an increased cognitive decline, a reduced mobility, or unavailability of transport. Subsequently, it may lessen the disparity of care for people with dementia living in rural and remote areas [31].

It must be noted that the guide is context-dependent, and the inequities of provision must be acknowledged. Depending on where the person with dementia and their family is residing, there may not be access to all of the music experiences presented in this guide. Furthermore, the access to the online-based music experiences is impacted by several factors, including caregiver support (where the person with dementia may not be able to use technology or at the later stage of their diagnosis), access to appropriate technological devices and internet connection, and socioeconomic status [96]. The presented document may be altered to match the availability of services to a specific region or organization to address this limitation. The applicability and usability of this document by people with dementia and their family caregivers at various stages of the dementia trajectory is recommended. The relevance of this guide in different cultural contexts and the health systems that exist within them is also an area for further exploration.

## 6. Conclusions

“Music has the capacity to be a means of connecting, communicating, companioning,” comforting, strengthening identity, and enriching the lives of people with dementia and their families who are living in the community [97], p. 42. This paper presents a guide supporting the selection of the most appropriate music experiences on their shared journey. The use of music in the dementia care pathway should not be limited to one profession or approach to practice. It is an interdisciplinary field, which encompasses a broad range of music experiences including but not limited to music therapy, community music, music medicine, and individualized resources used as a part of everyday life, including music listening, singing, or playing an instrument. Each of these music experiences plays a unique role in the person’s journey with dementia and should be integrated as a pathway of dementia care. This guide highlights that there is no “one size fits all” but rather that music experiences must be individually tailored to fit the person.

## Figures and Tables

**Table 1:** Terms Used in Literature Search.

Music Experiences	Dementia	Context
“Choir” OR “group singing” OR “Music” OR “music-based interventions” OR “music listening” OR “music activities” OR “musical instrument” OR “music therapy” OR “playlist” OR “singing”	“Alzheimer *” OR “cognitive impairment” OR “cognitive decline” OR “dementia” OR “older adults”	“Community” OR “community dwelling” OR “dwelling” OR “home” OR “home-based”

## Data Availability

The data presented in this study are available on request from the corresponding author. The data are not publicly available due to ensure confidentiality.

## Supplementary Materials

The following supporting information can be downloaded at: https://www.mdpi.com/article/10.3390/ijerph20105818/s1, Figure S1: First Draft of “Follow the Musical Road”.
