# Supplementary figures and images for: “Follow the Musical Road”: Selecting Appropriate Music Experiences for People with Dementia Living in the Community
Source: Int J Environ Res Public Health. 2023 May 13;20(10):5818. doi: 10.3390/ijerph20105818 (PMC10217950; doi:10.3390/ijerph20105818)

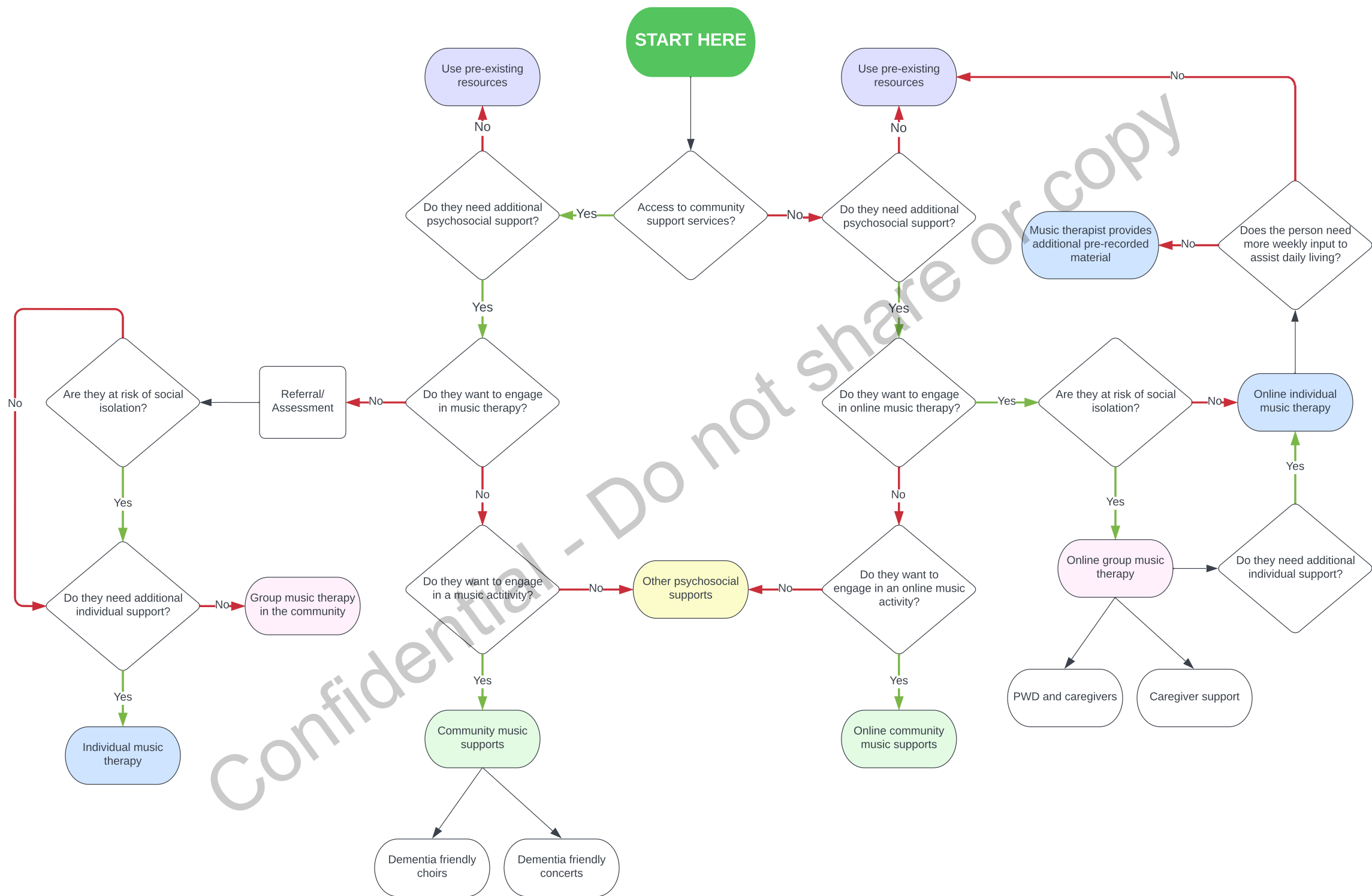

Supplement: Supplementary file 1 [file ijerph-20-05818-s001.zip › ijerph-2296764-supplementary.pdf]
